# Supplementary material for: Intermittent aerobic-resistance interval training versus continues aerobic training: Improvement in cardiac electrophysiologic and anthropometric measures in male patients post myocadiac infarction, a randomized control trial
Source: PLoS One. 2022 May 3;17(5):e0267888. doi: 10.1371/journal.pone.0267888 (PMC9064084; doi:10.1371/journal.pone.0267888)
Supplement: Attachment — Submitted filename: PONE-D-22-01160_reviewer.pdf [file pone.0267888.s002.pdf]

# PLOS ONE

## Intermittent Aerobic-Resistance Interval Training: improvement in cardiac electrophysiologic and cardio-metabolic measures, a randomized control trial

--Manuscript Draft--

| | |
|---|---|
| **Manuscript Number:** | PONE-D-22-01160 |
| **Article Type:** | Research Article |
| **Full Title:** | Intermittent Aerobic-Resistance Interval Training: improvement in cardiac electrophysiologic and cardio-metabolic measures, a randomized control trial |
| **Short Title:** | Interval training and cardiac electro measures |
| **Corresponding Author:** | Sharon Barak<br>Tel HaShomer Hospital: Sheba Medical Center at Tel Hashomer<br>Ramat Gan, None ISRAEL |
| **Keywords:** | Cardiac rehabilitation; Myocardial Infarction; circuit training; heart rate variability; Echocardiography. |
| **Abstract:** | Purpose: Exercise is a valuable intervention modality post-myocardial infarction (MI). Aerobic and resistance training are both commonly used separately in cardiac rehabilitation. However, the effect of aerobic interval exercise combined with alternating sets of resistance training (super-circuit training, SCT) on cardiac electrophysiologic and metabolic measures had not been thoroughly investigated. Aim: The primary objective of this study was to compare the effectiveness of moderate-intensity continuous-aerobic training (CAT) vs. SCT on cardiac electrical measures (resting electrocardiographic, ECG; and heart rate variability, HRV) in patients' post-MI presenting reduced left ventricular function. Second, to examine its effect on metabolic measures. Material and Methods: Twenty-nine men post-MI with reduced left ventricular function were assigned randomly to either 12 weeks of CAT (n = 15) or SCT (n = 14). CAT group performed moderate-intensity activity. SCT group performed high-intensity exercise, alternating between resistance and aerobic training. Differences between CAT and SCT groups were done using independent t-tests, paired t-tests and effect size (ES). Results: Participants in both groups improved their HRV measures (increase in HFnu; $p < 0.05$; ES > 0.51) and ECG (reduction in QT-dispersion; $p < 0.05$; ES > 0.51). Only the SCT group had significant improvements in waist circumference ($p < 0.05$). Conclusion: Exercise improves cardiac electrical measures post-MI. However, in comparison to CAT, SCT may yield greater metabolic changes. In order to have improvements in cardiac electrical stability, clinicians working with post-MI patients may use both CAT and SCT. However, SCT might result in greater improvements. |
| **Order of Authors:** | Horesh Dor-Haim, Ph.D |
| | Michal Horowitz |
| | Eldad Yaakobi |
| | Sara Katzburg, Ph.D |
| | Sharon Barak |
| **Additional Information:** | |
| **Question** | **Response** |
| **Financial Disclosure**<br><br>Enter a financial disclosure statement that describes the sources of funding for the work included in this submission. Review the submission guidelines for detailed requirements. View published research articles from *PLOS ONE* for specific | The author(s) received no specific funding for this work. |

*Powered by Editorial Manager® and ProduXion Manager® from Aries Systems Corporation*

| | |
|---|---|
| examples.<br><br>This statement is required for submission and **will appear in the published article** if the submission is accepted. Please make sure it is accurate.<br><br>**Unfunded studies**<br>Enter: *The author(s) received no specific funding for this work.*<br><br>**Funded studies**<br>Enter a statement with the following details:<br>• Initials of the authors who received each award<br>• Grant numbers awarded to each author<br>• The full name of each funder<br>• URL of each funder website<br>• Did the sponsors or funders play any role in the study design, data collection and analysis, decision to publish, or preparation of the manuscript?<br>• **NO** - Include this sentence at the end of your statement: *The funders had no role in study design, data collection and analysis, decision to publish, or preparation of the manuscript.*<br>• **YES** - Specify the role(s) played.<br><br>* typeset | |
| **Competing Interests**<br><br>Use the instructions below to enter a competing interest statement for this submission. On behalf of all authors, disclose any competing interests that could be perceived to bias this work—acknowledging all financial support and any other relevant financial or non-financial competing interests.<br><br>This statement is required for submission and **will appear in the published article** if the submission is accepted. Please make sure it is accurate and that any funding sources listed in your Funding Information later in the submission form are also declared in your Financial Disclosure statement. | The authors have declared that no competing interests exist. |

| | |
|---|---|
| View published research articles from *PLOS ONE* for specific examples.<br><br>**NO authors have competing interests**<br><br>Enter: *The authors have declared that no competing interests exist.*<br><br>**Authors with competing interests**<br><br>Enter competing interest details beginning with this statement:<br><br>*I have read the journal's policy and the authors of this manuscript have the following competing interests: [insert competing interests here]*<br><br>* typeset | |
| **Ethics Statement**<br><br>Enter an ethics statement for this submission. This statement is required if the study involved:<br><br>• Human participants<br>• Human specimens or tissue<br>• Vertebrate animals or cephalopods<br>• Vertebrate embryos or tissues<br>• Field research<br><br>Write "N/A" if the submission does not require an ethics statement.<br><br>General guidance is provided below. Consult the submission guidelines for detailed instructions. **Make sure that all information entered here is included in the Methods section of the manuscript.** | The study was approved by the Helsinki ethics committee, Hadassah medical center (0440-12-HMO, ClinicalTrials.gov Identifier: NCT01912690). All participants gave written informed consent. |

| | |
|---|---|
| **Format for specific study types**<br><br>**Human Subject Research (involving human participants and/or tissue)**<br>• Give the name of the institutional review board or ethics committee that approved the study<br>• Include the approval number and/or a statement indicating approval of this research<br>• Indicate the form of consent obtained (written/oral) or the reason that consent was not obtained (e.g. the data were analyzed anonymously)<br><br>**Animal Research (involving vertebrate animals, embryos or tissues)**<br>• Provide the name of the Institutional Animal Care and Use Committee (IACUC) or other relevant ethics board that reviewed the study protocol, and indicate whether they approved this research or granted a formal waiver of ethical approval<br>• Include an approval number if one was obtained<br>• If the study involved *non-human primates*, add *additional details* about animal welfare and steps taken to ameliorate suffering<br>• If anesthesia, euthanasia, or any kind of animal sacrifice is part of the study, include briefly which substances and/or methods were applied<br><br>**Field Research**<br><br>Include the following details if this study involves the collection of plant, animal, or other materials from a natural setting:<br>• Field permit number<br>• Name of the institution or relevant body that granted permission | |
| **Data Availability**<br><br>Authors are required to make all data underlying the findings described fully available, without restriction, and from the time of publication. PLOS allows rare exceptions to address legal and ethical concerns. See the PLOS Data Policy and FAQ for detailed information. | No - some restrictions will apply |

| | |
|---|---|
| A Data Availability Statement describing where the data can be found is required at submission. Your answers to this question constitute the Data Availability Statement and **will be published in the article**, if accepted.<br><br>**Important:** Stating 'data available on request from the author' is not sufficient. If your data are only available upon request, select 'No' for the first question and explain your exceptional situation in the text box.<br><br>Do the authors confirm that all data underlying the findings described in their manuscript are fully available without restriction? | |
| **Describe where the data may be found in full sentences. If you are copying our sample text, replace any instances of XXX with the appropriate details.**<br><br>• If the data are **held or will be held in a public repository**, include URLs, accession numbers or DOIs. If this information will only be available after acceptance, indicate this by ticking the box below. For example: *All XXX files are available from the XXX database (accession number(s) XXX, XXX.).*<br>• If the data are all contained **within the manuscript and/or Supporting Information files**, enter the following: *All relevant data are within the manuscript and its Supporting Information files.*<br>• If neither of these applies but you are able to provide **details of access elsewhere**, with or without limitations, please do so. For example:<br><br>*Data cannot be shared publicly because of [XXX]. Data are available from the XXX Institutional Data Access / Ethics Committee (contact via XXX) for researchers who meet the criteria for access to confidential data.*<br><br>*The data underlying the results presented in the study are available from (include the name of the third party* | data available on request from the autho |

| | |
|---|---|
| *and contact information or URL).*<br>• This text is appropriate if the data are owned by a third party and authors do not have permission to share the data.<br><br>* typeset | |
| Additional data availability information: | |

Manuscript Click here to access/download;Manuscript;Manuscript.docx



# Title page

**Title:** Intermittent Aerobic-Resistance Interval Training: improvement in cardiac electrophysiologic and cardio-metabolic measures, a randomized control trial

**Short title:** Interval training and cardiac electro measures

**Authors:** Horesh Dor-Haim[a], Michal Horowitz[b], Eldad Yaakobi[c], Sara Katzburg[ad], Sharon Barak[*e,f]

**Affiliations:** [a]O2 Health Promotion and Sports Medicine Department, Givat Ram, Jerusalem, Israel; [b]the laboratory of environmental physiology Department of Physiology, Faculty of Dentistry Hebrew University of Jerusalem, Hadassah Ein Kerem Campus Jerusalem, Israel; [c]The Sagol Center for Hyperbaric Medicine and Research, Yitzhak Shamir Medical Center - Be'er Ya'akov, Israel; [d]*Department of Developmental Biology and Cancer Research, Israel-Canada Medical Research Institute, Faculty of Medicine, Hebrew University of Jerusalem, Israel;* [e]The Edmond and Lily Safra Children's Hospital, the Chaim Sheba Medical Center, Ramat Gan, Israel; [f]Kaye Academic College of Education, Beer-Sheba, Israel.

# Abstract

**Purpose:** Exercise is a valuable intervention modality post-myocardial infarction (MI). Aerobic and resistance training are both commonly used separately in cardiac rehabilitation. However, the effect of aerobic interval exercise combined with alternating sets of resistance training (super-circuit training, SCT) on cardiac electrophysiologic and metabolic measures had not been thoroughly investigated. **Aim:** The primary objective of this study was to compare the effectiveness of moderate-intensity continuous-aerobic training (CAT) vs. SCT on cardiac electrical measures (resting electrocardiographic, ECG; and heart rate variability, HRV) in patients' post-MI presenting reduced left ventricular function. Second, to examine its effect on metabolic measures. **Material and Methods:** Twenty-nine men post-MI with reduced left ventricular function were assigned randomly to either 12 weeks of CAT (n = 15) or SCT (n = 14). CAT group performed moderate-intensity activity. SCT group performed high-intensity exercise, alternating between resistance and aerobic training. Differences between CAT and SCT groups were done using independent t-tests, paired t-tests and effect size (ES). **Results:** Participants in both groups improved their HRV measures (increase in HFnu; $p < 0.05$; ES > 0.51) and ECG (reduction in QT-dispersion; $p < 0.05$; ES > 0.51). Only the SCT group had significant improvements in waist circumference ($p < 0.05$). **Conclusion**: Exercise improves cardiac electrical measures post-MI. However, in comparison to CAT, SCT may yield greater metabolic changes. In order to have improvements in cardiac electrical stability, clinicians working with post-MI patients may use both CAT and SCT. However, SCT might result in greater improvements.


**Keywords:** Cardiac rehabilitation, myocardial infarction, circuit training, heart rate variability, Echocardiography.

# Introduction

Exercise training is considered an essential approach component for rehabilitation and secondary prevention of coronary heart disease [1] and usually consists of moderately intense continuous aerobic training (CAT) [2]. The benefits of CAT in cardiac patients are well established [2–4]. However, CAT leads to only a minor increase in muscle mass or strength and is usually associated with a more pronounced improvement in aerobic exercise capacity [5]. Thus, resistance training is part of every guideline for exercise-based cardiac rehabilitation [1]. There is evidence for the safety and efficacy of resistance training in cardiac patients [6,7]. For example, studies showed that appropriate resistance training induces metabolic, histochemical, and functional adaptations in skeletal muscles. Resistance training also increases muscle mass and strength effectively [8].

In cardiac patients, high-intensity interval training is safe, feasible, and is more effective than CAT in several outcome measures (e.g., peak oxygen consumption) [9,10]. Super-circuit training (SCT) is a novel type of training that involves two combined training modalities: resistance-training set simultaneously followed by an aerobic exercise interval. Studies on SCT showed that this type of training in healthy individuals increased strength and aerobic capacity. Compared to CAT, intensive SCT among healthy overweight middle-aged men led to a significant decrease in metabolic indices [8]. These results are of special interest in cardiac rehabilitation  as studies have shown that obesity can increase the risk of sudden death due to arrhythmic disorders [11] and changes in the autonomic system [12–14]. In a previous study, we compared the effects of CAT to SCT on men post-myocardial infarction (MI) with reduced left ventricle function. SCT yielded better benefits to the patient's mechanical cardiac function (left ventricle mechanical function). Moreover, compared to CAT, SCT yielded a better benefit to the

patient's fitness level, namely, aerobic capacity and strength [15]. However, the effects of CAT and SCT on cardiac electrical outcome measures [resting electrocardiograph (ECG) and heart rate variability (HRV)], were not analyzed. These non-invasive measures are of special interest as they correlate with patients' cardiovascular health and electrophysiological stability in individuals with cardiac conditions (e.g., left ventricle dysfunction) [16,17]. Since obesity is associated with adverse cardiac events [11], it was also vital to examine the CAT and SCT effects on metabolic-related measures.

The primary objective of the study was to compare the effectiveness of SCT vs. CAT on cardiac electrical outcome measures (i.e., resting ECG and HRV) in post-MI patients with reduced left ventricle function. Our second objective was to compare the effects of these two regimes on patient's metabolic measures (body mass index, BMI; and waist circumference).

# Materials and Methods

## Study participants

Post-MI male patients were referred to the cardiac rehabilitation center at Hadassah Mt. Scopus 6±10 weeks' post-hospitalization due to acute MI. Inclusion criteria: 1) echo testing exhibited reduced left ventricle function (ejection fraction < 45%), 2) patients were able to attend regularly a supervised exercise program. Exclusion criteria: 1) chronic atrial fibrillation, 2) severe valvular disease, 3) angina or peripheral arterial occlusive disease, and 4) cerebrovascular or musculoskeletal disease-preventing exercise testing or training. The study was approved by the Helsinki ethics committee, Hadassah medical center (0440-12-HMO, ClinicalTrials.gov Identifier: NCT01912690). All participants gave written informed consent.

## Study procedures

Participants were randomly assigned (sealed envelope method) to either twice a week CAT or SCT by cardiac rehabilitation staff who were not involved in the research study. The technical work and analysis of the results were blinded. The care providers were not blinded. During the trial, drug therapy remained unchanged, and patients with type 2 diabetes, or hypertensive, were not regulated in their drug therapy dosage during the 12-week intervention. A participant was deducted from the study if he developed adverse effects such as chest tightness, ECG changes, or severe arrhythmias.

Patients in both groups started each training session with five minutes warm-up, followed by either CAT or SCT. Throughout the training sessions, exercise intensity was defined using heart rate measurement (Polar Electro, Kempele, Finland; or Nihon Kohden ECG telemetry) and calculation of heart rate reserve (i.e., maximal heart rate – resting heart rate). Participants' maximal heart rate was established using a baseline graded exercise tolerance test. At the end of the exercise, both groups conducted five minutes of gradual cool down.

CAT group participants exercised continually at 60% to 70% of their heart rate reserve using a treadmill (TechnoGym) and lower and upper extremity cycle ergometer (Star Terk). A modified Borg 1-to-10 scale was used to assess the rate of perceived exertion, during and after each training session. Speed and inclination of the treadmill or resistance and cadence of the cycle ergometer were adjusted continuously, to ensure that every training session was carried out at the assigned heart rate reserve throughout the training period (fig 1).

**Figure 1. Super-circuit training and continuous-aerobic training – training program (adapted from Dor-Haim et al., 2018)[15] -** Continues aerobic training group conducted only continues aerobic training and super-circuit training group conducted both aerobic and strength training.

The SCT group performed high-intensity exercise, alternating between resistance and aerobic training. One resistant training set was followed by three minutes of aerobic exercises and a resting period, repeated eight times. Each exercise consisted of one set of 15 repetitions on a Cybex machine. In the first two weeks of the program, the training intensity was light - 30% of 1-repetition maximum, and progressively increased to 50% of 1-repetition maximum. Participants had to maintain appropriate lifting techniques, not hold their breath to avoid the Valsalva maneuver, and carefully alternate positions to adapt to the blood pressure orthostatic changes. The aerobic intensity was determined at 75% to 85% of heart rate reserve. Resting periods between intervals was minimal, and gradually decreased from two minutes in the first two weeks to 1.5 minutes in weeks 3-6 and 1 minute in weeks 7-12 (fig 1).

For a more detailed description of the study design and exercise protocol, refer to Dor-Haim et al (2018) [15].

# Outcome measures

All outcome measures were evaluated at baseline and post-12 weeks of the exercise training.

## Cardiac electrical measures - primary outcome measures

1. Resting electrocardiogram (ECG) – Twelve lead resting ECG for a duration of 10 minutes was recorded before the graded exercise tolerance test (Cardio scape PC ECG,

version 3.1) using a fixed speed of 25 mm/sec and standardization of 1 mm as 1 millivolt throughout the measurement. QT interval was measured from the QRS complex onset point to the T wave offset point (the return of T wave to baseline). QT-dispersion (QTd) and Corrected QTd (QTcd) was calculated as the difference between the maximum and minimum QT intervals for any of the 12 leads [18].

2. Heart rate variability (HRV): Measurements of heart rate (Cardio scape PC ECG, version 3.1) were performed at rest. For analysis and calculation of HRV, the Kubios HRV software 2.0 was used [19]. The data were visually examined for quality control. Periods of excessive noise were removed from the analysis. Spectral analysis was used to calculate high-frequency (0.15–0.40 Hz) and low frequency (0.04–0.15 Hz) normalized power (HFnu and LFnu, respectively) using Fast Fourier Transformation. The ratio of low-to-high frequency (LF/HF) band powers was evaluated. A frequency-domain analysis method was selected for comparison to time-domain measures, as frequency-based analyses provide a more accurate interpretation of parasympathetic and sympathetic influences [16,20].

## Metabolic measures

Two metabolic parameters were measured: body mass index (BMI; weight in kg/height in $m^2$) and waist circumference (measured 2.5 centimeters above the umbilicus) [21].

## Data analysis

Differences between CAT and SCT groups in the various outcome measures at both pre and post-tests were examined using independent t-tests. To evaluate within-group changes from pre-to-post-test in the various outcome measures, paired t-tests were conducted (alpha level $p<0.05$, adjusted to 0.025 using the Bonferroni procedure). For HRV measurements, alpha level

was adjusted to 0.016 (0.05/3= 0.016). Effect sizes (ES) using Cohen's *d* [22] were also calculated in order to quantify the degree of change in each study group. A correction for the dependence among means was done using the correlations between the two means following Morris and DeShon's equation [23]. In general, values smaller and equal to 0.20 were considered trivial ES, values between 0.21 and 0.50 as small ES, values 0.51-0.80 as moderate ES, and values greater than 0.80 as large ES [22].

A posthoc power analysis was conducted using the main outcome measures, mainly, ECG and HRV. The average ES of these variables in the CAT and SCT groups were 1.78 and 0.78, respectively. Using posthoc power analysis for the ESs, α =0.05, and the study's sample size, the power to detect differences between two dependent variables (matched pairs) was 0.90 and 0.80, respectively. The mean ES difference between the groups was 0.78. Given this ES, α = 0.05, and the study's sample size, the study's power to detect between-group differences (independent t-test) was 0.78. Power analysis calculations were done using G*Power (version 3.0.10).

# Results

## Study participants

Fifty-eight patients were referred to the cardiac rehabilitation center. Ten participants declined to participate or did not meet the inclusion criteria. Forty-eight participants (mean age = 59.14 years old + 8.92; range: 42.00-75.00) were assigned randomly to the CAT (n=26) or SCT (n=22). In the CAT group, post-test data were available for 15 participants. In the SCT group, post-test data were available for 14 participants. For study participants flow-chart, refer to fig 2.

**Figure 2. Study participants' flow chart (adapted from Dor-Haim et al., 2018)[15] -** Forty-eight participants were assigned randomly to the continues aerobic training (n=26) or super circuit training (n=22). In the continues aerobic training group, 19 participants completed the program. Post-test data were available for 15 participants. In the super circuit training group, 16 participants completed the program, post-test data were available for 14 participants.

## Cardiac electrical measures

The CAT group showed significant differences from pre to post-test in two HRV measures (increased HFnu and decreased LF nu; $p < 0.016$, fig 3a) and in QT ECG measures (i.e., decreased QTd and QTcd; $p < 0.025$, fig 3b). The SCT group yielded a significant increase in HFnu ($p < 0.016$, fig 3a). The SCT group did not demonstrate significant changes in LFnu (fig 3a). The SCT group showed significant changes in the two ECG measures ($p < 0.025$, fig 3b). Regarding ES, the changes observed in both study groups were moderate-to-large (Cohen's $d > 0.51$, table 1). No significant between-group changes were found in the cardiac electrical measures, except for LFnu that in comparison to the SCT group, was significantly lower in the CAT group at pre-test (p=0.006, fig 3a and 3b).

Table 1: Continuous aerobic training and super-circuit training effect sizes

| **Primary and secondary outcome measures** | **Cohen's *d* effect size** |
|---|---|

| | Training group | Continues aerobic training | Super-circuit training |
|---|---|---|---|
| Resting electrocardiograph | QT dispersion | -1.24 | 0.91 |
| | Corrected QT dispersion | -1.22 | -0.83 |
| Heart rate variability | $HF_{nu}$ | 1.839 | 1.07 |
| | $LF_{nu}$ | -3.73 | -0.51 |
| | LF/HF | -0.90 | -0.55 |
| Metabolic measures | Body mass index | -0.08 | -0.34 |
| | Waist circumference (cm) | -0.06 | -0.55 |

Note: Cohen's d calculation: mean Δ/standard deviation average from two means. Dark gray cells -moderate and large differences (Cohen's $d \geq 0.51$). Light gray cells - small differences (Cohen's $d$ = 0.21-0.50). White cells - trivial differences (Cohen's $d \leq 0.20$). *Cohen's *d* is based on a single pooled standard deviation. Cohen's *d* was corrected for dependence between means, using Morris and DeShon's equation.[23]

**Figure 3: Within and between-group differences cardiac electrical measures.**

**3a - heart rate variability. 3b – electrocardiograph** - Analysis level of significance was set at 0.05 and adjusted to 0.016 in heart rate variability measures (i.e., HF, LF, and LF/HF) and 0.025 in electrocardiograph measures (i.e., QT dispersion and corrected QT dispersion), using the Bonferroni procedure). The central box represents the values from the lower to upper quartile (25 to 75 percentile). The vertical line extends from minimum to maximum values, excluding the outside values displayed as separate points; *significant within-group changes from pre to post-

test; [a]between-group differences - significantly different than CAT group; [b]between-group differences - significantly different than SCT group.

## Metabolic measures

No statistical significant between-group differences in BMI were observed in at both pre and post-tests. Similarly, in both groups participants did not present a statistically significant change from pre-to-post-test in BMI. Waist circumference of SCT groups was statistically significantly lower than this of CAT group in both pre-and-posttest. Moreover, only the SCT group presented a significant reeducation in waist circumference from pre-to-posttest ($p<0.025$; ES = -0.55). For additional information, refer to tables 1 and 2.

Table 2: Metabolic measures: within and between group differences.

| Variables | | Continues aerobic training (n=15) | | | Super-circuit training (n=14) | | | Between-groups analysis | |
|---|---|---|---|---|---|---|---|---|---|
| | | **Pre-test: mean (SD)** | **Post-test: mean (SD)** | **Statistic t (p value)** | **Pre-test: mean (SD)** | **Post-test: mean (SD)** | **Statistic t (p value)** | **Pre-test: statistic t (p value)** | **Post-test: statistic t (p value)** |
| Metabolic Measures | Body mass index | 29.35 (3.81) | 29.04 (3.65) | -1.488 (0.164) | 28.14 (2.90) | 27.14 (2.73) | -2.72 (0.026) | -0.789 (0.439) | -1.305 (0.207) |
| | Waist circumference (cm) | 107.56 (7.36) | 107.12 (7.88) | -1.01 (0.302) | 98.66 (7.17) | 94.66 (6.94) | -6.35 (0.0002) * | -2.59 (0.019) | -3.55 (0.002) * |

Notes: SD, standard deviation; * significant within or between-group differences at the $p < 0.05$ (alpha level of body composition measurements was adjusted to 0.025 using the Bonferroni procedure.

# Discussion

This study's primary objective was to evaluate the implications of two-exercise regimes post-MI in patients with RVLF on electrocardiographic measures (resting ECG and HRV). The second objective was to compare their effect on metabolic measures. In our previous study [15], SCT was more effective than CAT in enhancing cardiac mechanical systolic (ejection fraction) and diastolic function (mitral inflow E velocity to tissue Doppler Le′ ratio). Resting ECG measure such as QTd and HRV are key electrophysiological markers post-MI in ischemic patients. These measures are related to electrical remodeling of the heart post-MI, arrhythmias such as atrial fibrillation [24], while such modeling in the ventricle may cause potentially lethal ventricular arrhythmias [25]. Therefore, the effects of CAT and SCT on electrocardiograph measures were essential for investigation.

The results presented here indicate an improvement in both QTd and HRV by both CAT and SCT regimes. More specifically, in both groups, QTd and QTdc decreased ($p<0.01$). Moreover, both groups showed a significant increase in HF spectral component, attributed to an increase in parasympathetic tone and antiarrhythmic protection [26]. Our results are consistent with other studies indicating the benefits of exercise training for the heart's electrical stability post-MI [17]. The beneficial effects of both CAT and SCT on ECG and HRV measures are encouraging as such improvements are associated with reduced cardiovascular disease burden and mortality. Other clinical and animal studies showed that QTd and HRV are independent non-invasive markers for ventricular arrhythmia and sudden cardiac death following MI [27,28]. QTd also serves as a marker for electrical inhomogeneity during myocardial repolarization. In addition, HRV is related to autonomic nervous system regulation. In MI survivors, lower HRV

was associated with the remodeling of the autonomic nervous system [29] and an increased risk of tachyarrhythmia [30]. Therefore, improved HRV and QTd in the current study by safe exercise protocol may elicit favorable cardiac electrical reversed remodeling among patients' post-MI with reduced left ventricle function.

Few studies showed a deleterious arrhythmogenic effect of exercise, mostly in vigorous exercise [31,32]. In the current study, we demonstrated that both exercise regimes resulted in an improvement in electrical markers. However, the SCT method resulted in a better cardiac intrinsic recovery, and thus, may yield a better prognosis post-MI. Animal studies also demonstrated that increased exercise intensity improves cardiovascular electrical stability in a dose-response relationship [33]. In the current study, the only difference between the two training regimes was found in the LF component, which was decreased in the CAT group but not in the SCT group. The LF component represents the interaction of the sympathetic and parasympathetic nervous systems. Exercise training induces adaptations in HRV outcomes with a shift of autonomic balance toward higher parasympathetic activity, consistent with improved cardiac health [16]. The decrease in the LF component is commonly observed in MI survivors and is attributed to increased sympathetic tone and increased risk of sudden death [34]. Hence, from the electrophysiological aspect attenuated LF reduction seen only in the SCT group may represent a relatively improved clinical reaction

Both training groups did not show significant changes in pre-post BMI, suggesting that participants did not lose or gain weight. However, the SCT group presented significant reductions in waist circumference. These results showed that only the SCT group decreased their abdominal visceral fat [35,36], which is one component of metabolic syndrome [37]. Reduction of body fat without losing weight indicates a gain in muscle mass at the expense of fat mass loss

[38]. Compared to subjects with high muscle/low fat, the risk of arrhythmia due to cardiac intrinsic electrical instability is significantly higher in people with a high fat /low muscle ratio [39]. It is also important to note that abnormal autonomic regulation is prevalent in metabolic patients. For example, several HRV studies showed abnormalities in autonomic nervous control in obese and overweight subjects [40,41]. In overweight individuals, a sympathovagal imbalance due to increased sympathetic activity and its association with visceral fat was observed [42]. In another 6-month study, aerobics alone was compared to a combined aerobic and resistance training. Both regimes significantly decreased abdominal visceral fat, but combined aerobic and resistance training was more effective [36].

The current study was subject to several limitations. First, the generalizability of the results is in question owing to the single center and the small sample size in each study group. Second, the study period of three months is too short for concluding the prognostic indicators for the two exercise groups. Prolonged research and follow-up are required to allow firm conclusions regarding re-hospitalizations and mortality.

# Conclusions

In conclusion, the current study showed that electrocardiographic measures post-MI stand to benefit from both training regimes, namely, CAT and SCT. Nevertheless, in comparison to aerobic training alone (i.e., CAT), SCT may yield better benefits to autonomic balance and metabolic measures. Considering the effect of exercise on MI patients, it is vital to introduce

novel training modalities that may enhance the central cardiac recovery and peripheral metabolic health-related factors post-MI.

## References

1. Leon AS, Franklin BA, Costa F, Balady GJ, Berra KA, Stewart KJ, Thompson PD, Williams MA, Lauer MS, American Heart Association, et al. Cardiac rehabilitation and secondary prevention of coronary heart disease: an American Heart Association scientific statement from the Council on Clinical Cardiology (Subcommittee on Exercise, Cardiac Rehabilitation, and Prevention) and the Council on Nutrition, Physical Activity, and Metabolism (Subcommittee on Physical Activity), in collaboration with the American association of Cardiovascular and Pulmonary Rehabilitation. Circulation. 2005 January 25;111:369–376.
2. Davies EJ, Moxham T, Rees K, Singh S, Coats AJS, Ebrahim S, Lough F, Taylor RS. Exercise training for systolic heart failure: Cochrane systematic review and meta-analysis. European Journal of Heart Failure. 2010 July;12:706–715.
3. Adachi H, Koike A, Obayashi T, Umezawa S, Niwa A, Marumo F, Hiroe M. Does appropriate endurance exercise training improve cardiac function in patients with prior myocardial infarction? European Heart Journal. 1996 October 2;17:1511–1521.
4. Adamopoulos S, Coats AJS, Brunotte F, Arnolda L, Meyer T, Thompson CH, Dunn JF, Stratton J, Kemp GJ, Radda GK, et al. Physical training improves skeletal muscle metabolism in patients with chronic heart failure. Journal of the American College of Cardiology. 1993 April;21:1101–1106.
5. Frankel JE, Bean JF, Frontera WR. Exercise in the Elderly: Research and Clinical Practice. Clinics in Geriatric Medicine. 2006 May;22:239–256.
6. Vanhees L, Geladas N, Hansen D, Kouidi E, Niebauer J, Reiner Ž, Cornelissen V, Adamopoulos S, Prescott E, Börjesson M. Importance of characteristics and modalities of

physical activity and exercise in the management of cardiovascular health in individuals with cardiovascular risk factors: recommendations from the EACPR (Part II). Eur J Prev Cardiolog. 2012 October;19:1005–1033.

7. Bjarnason-Wehrens B, Mayer-Berger W, Meister ER, Baum K, Hambrecht R, Gielen S. Recommendations for resistance exercise in cardiac rehabilitation. Recommendations of the German Federation for Cardiovascular Prevention and Rehabilitation. European Journal of Cardiovascular Prevention & Rehabilitation. 2004 August;11:352–361.
8. Paoli A, Pacelli F, Bargossi AM, Marcolin G, Guzzinati S, Neri M, Bianco A, Palma A. Effects of three distinct protocols of fitness training on body composition, strength and blood lactate. J Sports Med Phys Fitness. 2010 March;50:43–51.
9. Kessler HS, Sisson SB, Short KR. The Potential for High-Intensity Interval Training to Reduce Cardiometabolic Disease Risk: Sports Medicine. 2012 June;42:489–509.
10. Smart NA, Steele M. A Comparison of 16 Weeks of Continuous vs Intermittent Exercise Training in Chronic Heart Failure Patients: continuous vs intermittent exercise training in heart failure patients. Congestive Heart Failure. 2012 July;18:205–211.
11. Tavora F, Zhang Y, Zhang M, Li L, Ripple M, Fowler D, Burke A. Cardiomegaly is a common arrhythmogenic substrate in adult sudden cardiac deaths, and is associated with obesity. Pathology. 2012 April;44:187–191.
12. McKelvie RS, Teo KK, McCartney N, Humen D, Montague T, Yusuf S. Effects of exercise training in patients with congestive heart failure: A critical review. Journal of the American College of Cardiology. 1995 March;25:789–796.

13. Triggiani AI, Valenzano A, Trimigno V, Di Palma A, Moscatelli F, Cibelli G, Messina G. Heart rate variability reduction is related to a high amount of visceral adiposity in healthy young women Xu S-Z, editor. PLoS ONE. 2019 September 25;14:e0223058.
14. Spraul M, Ravussin E, Fontvieille AM, Rising R, Larson DE, Anderson EA. Reduced sympathetic nervous activity. A potential mechanism predisposing to body weight gain. J Clin Invest. 1993 October 1;92:1730–1735.
15. Dor-Haim H, Barak S, Horowitz M, Yaakobi E, Katzburg S, Swissa M, Lotan C. Improvement in cardiac dysfunction with a novel circuit training method combining simultaneous aerobic-resistance exercises. A randomized trial Earnest CP, editor. PLoS ONE. 2018 January 29;13:e0188551.
16. Anon. Heart rate variability: standards of measurement, physiological interpretation and clinical use. Task Force of the European Society of Cardiology and the North American Society of Pacing and Electrophysiology. Circulation. 1996 March 1;93:1043–1065.
17. Fujimoto S, Uemura S, Tomoda Y, Yamamoto H, Matsukura Y, Horii M, Iwamoto E, Hashimoto T, Dohi K. Effects of Exercise Training on the Heart Rate Variability and QT Dispersion of Patients With Acute Myocardial Infarction. Jpn Circ J. 1999;63:577–582.
18. Kiani A, Rafieyian S, Roodpeyma S, Sefidgarnia M. The Relationship between Coronary Artery Aneurysm and QT Interval Dispersion in Acute Phase of Kawasaki Disease. Iran J Pediatr. 2011 June;21:220–224.
19. Tarvainen MP, Niskanen J-P, Lipponen JA, Ranta-aho PO, Karjalainen PA. Kubios HRV – Heart rate variability analysis software. Computer Methods and Programs in Biomedicine. 2014 January;113:210–220.

20. Berntson GG, Thomas Bigger J, Eckberg DL, Grossman P, Kaufmann PG, Malik M, Nagaraja HN, Porges SW, Saul JP, Stone PH, et al. Heart rate variability: Origins, methods, and interpretive caveats. Psychophysiology. 1997 November;34:623–648.

21. R. S. Guerra TFA EA Marques, J Mota y M[a]T Restivo,-. Anatomical location for waist circumference measurement in older adults; a preliminary study. Nutricion Hospitalaria. 2012:1554–1561.

22. Cohen J. Statistical power analysis for behavioral sciences. Revised ed. New York: Academic Press; 1977.

23. Morris SB, DeShon RP. Combining effect size estimates in meta-analysis with repeated measures and independent-groups designs. Psychological Methods. 2002;7:105–125.

24. Carlsson L, Duker G, Jacobson I. New pharmacological targets and treatments for atrial fibrillation. Trends in Pharmacological Sciences. 2010 August;31:364–371.

25. Cutler MJ, Jeyaraj D, Rosenbaum DS. Cardiac electrical remodeling in health and disease. Trends in Pharmacological Sciences. 2011 March;32:174–180.

26. Billman GE. Cardiac autonomic neural remodeling and susceptibility to sudden cardiac death: effect of endurance exercise training. American Journal of Physiology-Heart and Circulatory Physiology. 2009 October;297:H1171–H1193.

27. Shekha K, Ghosh J, Thekkoott D, Greenberg Y. Risk stratification for sudden cardiac death in patients with non-ischemic dilated cardiomyopathy. Indian Pacing Electrophysiol J. 2005 April 1;5:122–138.

28. Malliani A, Lombardi F, Pagani M, Cerutti S. Power spectral analysis of cardiovascular variability in patients at risk for sudden cardiac death. J Cardiovasc Electrophysiol. 1994 March;5:274–286.

29. Besnier F, Labrunée M, Pathak A, Pavy-Le Traon A, Galès C, Sénard J-M, Guiraud T. Exercise training-induced modification in autonomic nervous system: An update for cardiac patients. Annals of Physical and Rehabilitation Medicine. 2017 January;60:27–35.

30. Sessa F, Anna V, Messina G, Cibelli G, Monda V, Marsala G, Ruberto M, Biondi A, Cascio O, Bertozzi G, et al. Heart rate variability as predictive factor for sudden cardiac death. Aging (Albany NY). 2018 February 23;10:166–177.

31. La Gerche A, Burns AT, Mooney DJ, Inder WJ, Taylor AJ, Bogaert J, MacIsaac AI, Heidbüchel H, Prior DL. Exercise-induced right ventricular dysfunction and structural remodelling in endurance athletes. European Heart Journal. 2012 April;33:998–1006.

32. Benito B, Gay-Jordi G, Serrano-Mollar A, Guasch E, Shi Y, Tardif J-C, Brugada J, Nattel S, Mont L. Cardiac Arrhythmogenic Remodeling in a Rat Model of Long-Term Intensive Exercise Training. Circulation. 2011 January;123:13–22.

33. Dor- Haim H, Lotan C, Horowitz M, Swissa M. Intensive Exercise Training Improves Cardiac Electrical Stability in Myocardial- Infarcted Rats. JAHA [Internet]. 2017 July [cited 2021 November 4];6. Available from: https://www.ahajournals.org/doi/10.1161/JAHA.117.005989

34. Guzzetti S, Rovere MTL, Pinna GD, Maestri R, Borroni E, Porta A, Mortara A, Malliani A. Different spectral components of 24 h heart rate variability are related to different modes of death in chronic heart failure. European Heart Journal. 2005 February 1;26:357–362.

35. Tremblay A, Simoneau J-A, Bouchard C. Impact of exercise intensity on body fatness and skeletal muscle metabolism. Metabolism. 1994 July;43:814–818.

36. Park S-K, Park J-H, Kwon Y-C, Kim H-S, Yoon M-S, Park H-T. The effect of combined aerobic and resistance exercise training on abdominal fat in obese middle-aged women. J Physiol Anthropol Appl Human Sci. 2003 May;22:129–135.

37. Grundy SM, Neeland IJ, Turer AT, Vega GL. Waist Circumference as Measure of Abdominal Fat Compartments. Journal of Obesity. 2013;2013:1–9.

38. Srikanthan P, Karlamangla AS. Muscle Mass Index As a Predictor of Longevity in Older Adults. The American Journal of Medicine. 2014 June;127:547–553.

39. Srikanthan P, Horwich TB, Tseng CH. Relation of Muscle Mass and Fat Mass to Cardiovascular Disease Mortality. The American Journal of Cardiology. 2016 April;117:1355–1360.

40. Schnabel RB, Aspelund T, Li G, Sullivan LM, Suchy-Dicey A, Harris TB, Pencina MJ, D'Agostino RB, Levy D, Kannel WB, et al. Validation of an Atrial Fibrillation Risk Algorithm in Whites and African Americans. Arch Intern Med [Internet]. 2010 November 22 [cited 2021 November 4];170. Available from: http://archinte.jamanetwork.com/article.aspx?doi=10.1001/archinternmed.2010.434

41. Frost L, Hune LJ, Vestergaard P. Overweight and obesity as risk factors for atrial fibrillation or flutter: the Danish Diet, Cancer, and Health Study. Am J Med. 2005 May;118:489–495.

42. Chintala KK, Krishna BH, N MR. Heart rate variability in overweight health care students: correlation with visceral fat. J Clin Diagn Res. 2015 January;9:CC06-08.

Figure 1

Click here to access/download;Figure;Figure_1.tiff

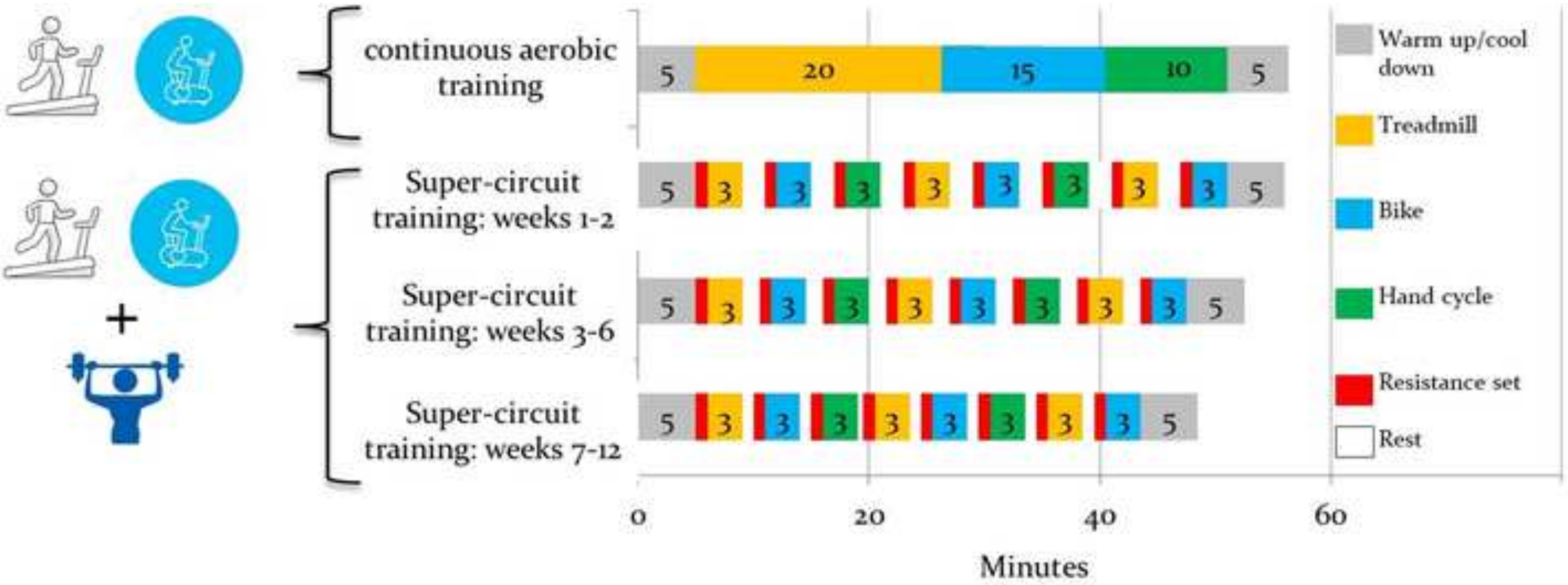

Figure 2 Click here to access/download;Figure;Figure_2.tif

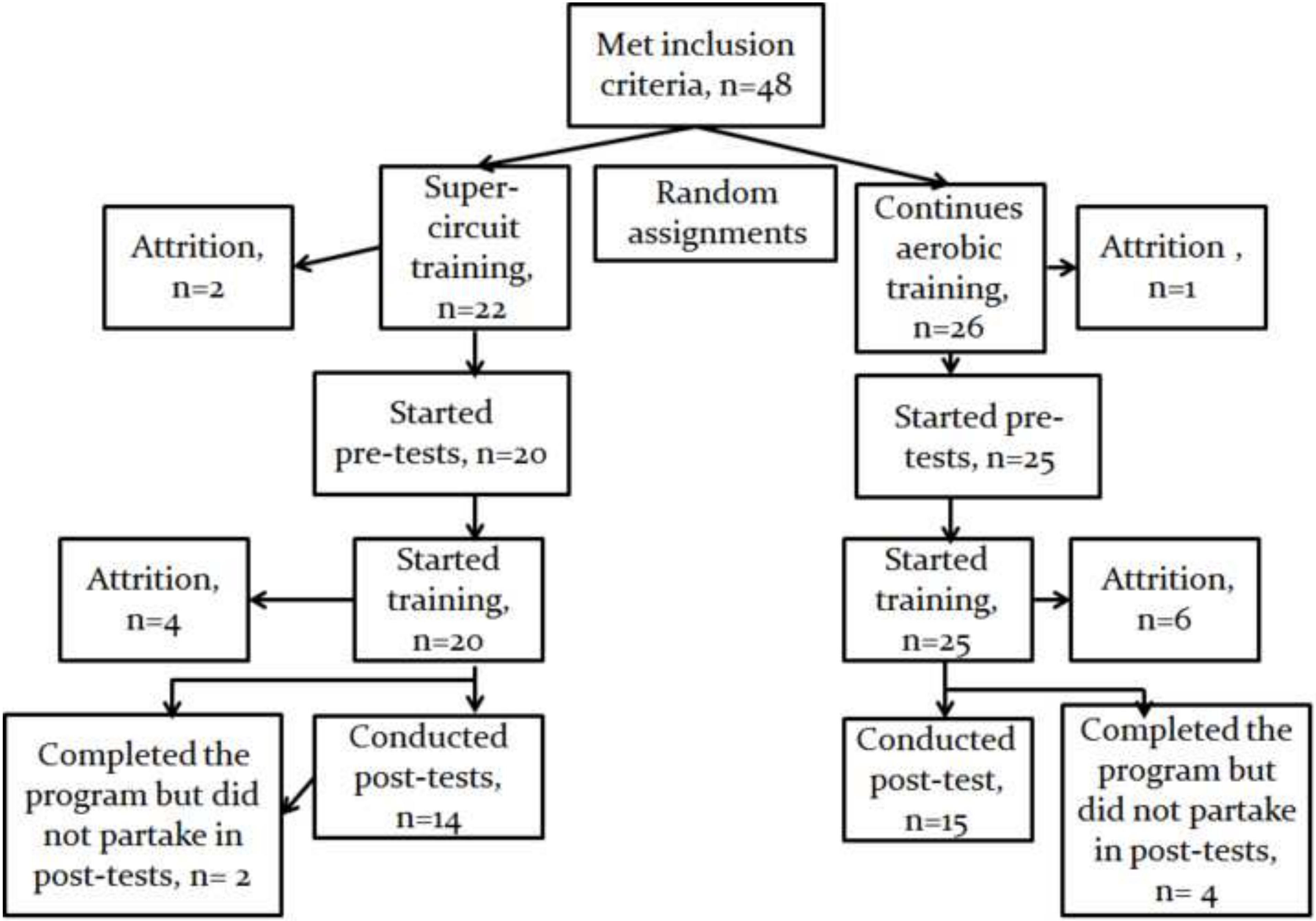

Figure 3

Click here to access/download;Figure;Figure_3.tif

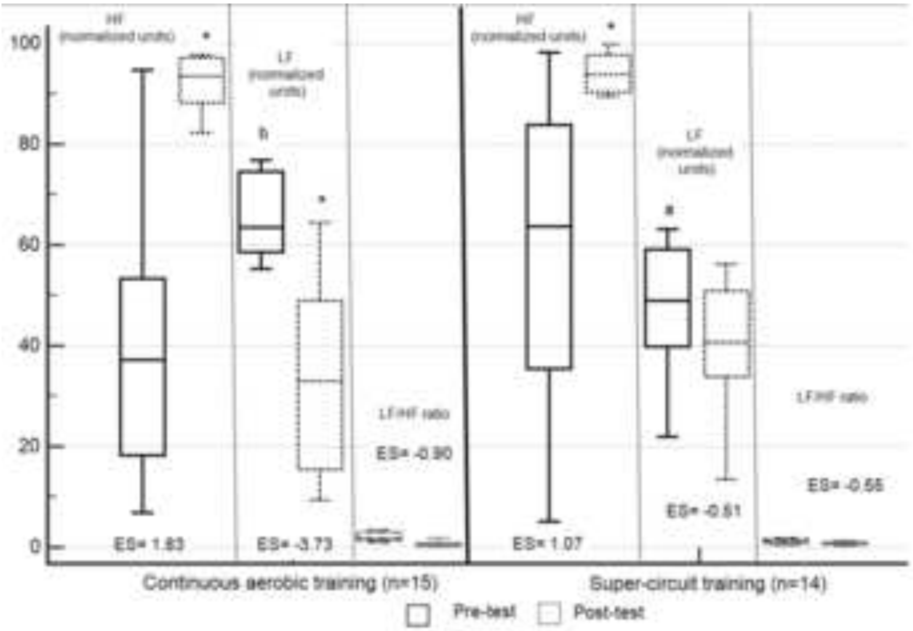

Figure 3a

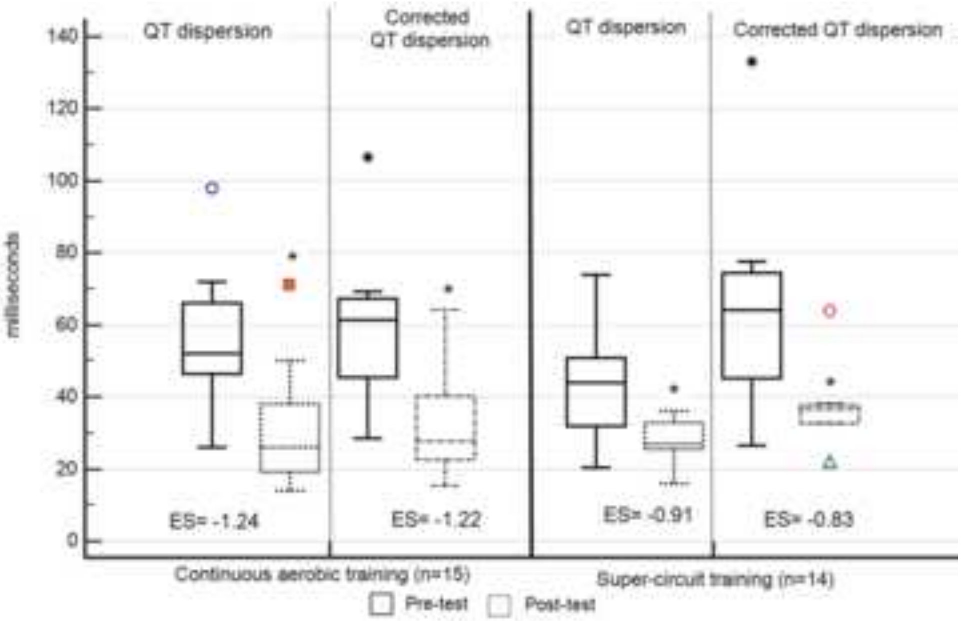

Figure 3b

Original Figure 1

Click here to access/download
**Supporting Information**
Original_Figure_1.png

Original Figure 2

Click here to access/download
**Supporting Information**
Original Figure 2.png

Copy of related work

Click here to access/download
**Supporting Information**
Copy of related work.pdf
