# Supplementary material for: Intermittent aerobic-resistance interval training versus continues aerobic training: Improvement in cardiac electrophysiologic and anthropometric measures in male patients post myocadiac infarction, a randomized control trial
Source: PLoS One. 2022 May 3;17(5):e0267888. doi: 10.1371/journal.pone.0267888 (PMC9064084; doi:10.1371/journal.pone.0267888)
Supplement: Attachment — Submitted filename: Response to reviewers_second.docx [file pone.0267888.s004.docx]

**Response to reviewers – second**

**PONE-D-22-01160R1**

Intermittent aerobic-resistance interval training versus continues aerobic training: improvement in cardiac electrophysiologic and cardio-metabolic measures in male patients post myocadiac infarction, a randomized control trial

**Reviewer 1**

Thank you very much for your feedback. We are happy that we were able to appropriately response to all your concerns. Your valuable comments helped improving the manuscript.

**Reviewer 2**

1. **IN HRV, Correct Normalized power to normalized unit (nu)**

Corrected.

2. **Metabolic measures, I suggest changing it into "anthropometric measures" which is more common and describing the measures used**.

Thank you very much for this comment. We changed in the entire manuscript "metabolic" to "anthropometric" except for places that cite from the literature references specifically related to metabolic measures.

3. **It caught my attention one of the responses to reviewers that minimum EF was not mentioned, and the authors answered that inclusion criteria is based on NYHA. Does the author suggest that this classification is safer than including EF?**

We used the NYHA classification as an inclusion criteria as it commonly used as a fundamental tool for risk stratification of heart failure and determines clinical trial eligibility and candidacy for drugs and devices. We did not try to suggest that this classification is safer than including EF. We rephrased the inclusion criteria to better explain the reason for using the NYHA.

Caraballo C, Desai NR, Mulder H, Alhanti B, Wilson FP, Fiuzat M, Felker GM, Piña IL, O’Connor CM, Lindenfeld J, et al. Clinical Implications of the New York Heart Association Classification. JAHA. 2019 December 3;8:e014240.

4. **Thanks for adding information about the medication and co-morbidities, but it would be better to include it in a table with the common demographical data such as age, weight height, BMI**

We created a new table, table 1. The table has information regarding the 2 study's groups' demographic and clinical characteristics. We also added between-group analyses for both continues and categorical variables. We did not put in the table BMI as BMI is one of the study's outcome measures and it appears with the other outcome measures in tables 2 and 3.

5. **Modalities that may enhance the "intrinsic" cardiac recovery and peripheral metabolic health-related factors post MI - what does the author mean with intrinsic, if the author agree on "parasympathetic recovery" then I would use Extrinsic instead of intrinsic**

Thank you for this comment. We reread the sentence and indeed it is not clear and accurate enough. We rephrased the sentence:

**"Considering the effect of exercise on MI patients, it is vital to introduce novel training modalities that may enhance autonomic balance, intrinsic myocardial recovery and health related anthropometric factors post-MI.**"

**Reviewer 3**

Thank you very much for your time reviewing the manuscript. We are happy that we were able to appropriately response to all your concerns. Your valuable comments helped improving the manuscript.
